# Scoping review of sexual and reproductive healthcare for men in the MENA (Middle East and North Africa) region: a handful of paradoxes?

**DOI:** 10.1186/s12889-022-14716-2

**Published:** 2023-03-27

**Authors:** Walid El Ansari, Mohamed Arafa, Haitham Elbardisi, Ahmad Majzoub, Mohammed Mahdi, Ahmed Albakr, Khalid AlRumaihi, Abdulla Al Ansari

**Affiliations:** 1 Department of Surgery, Hamad Medical Corporation, Doha, Qatar; 2 College of Medicine, Qatar University, Doha, Qatar; 3 Weill Cornell Medicine – Qatar, Doha, Qatar; 4 Urology Department, Hamad Medical Corporation, Doha, Qatar; 5 Andrology Department, Cairo University, Cairo, Egypt

**Keywords:** Public health, Sexual dysfunction, Sexual medicine, Reproductive healthcare, Middle East and North Africa

## Abstract

**Background:**

No study appraised the knowledge gaps and factors impacting men’s sexual and reproductive health (SRH) in MENA (Middle East and North Africa). The current scoping review undertook this task.

**Methods:**

We searched PubMed and Web of Science (WoS) electronic databases for original articles on men’s SRH published from MENA. Data was extracted from the selected articles and mapped out employing the WHO framework for operationalising SRH. Analyses and data synthesis identified the factors impacting on men’s experiences of and access to SRH.

**Results:**

A total of 98 articles met the inclusion criteria and were included in the analysis. The majority of studies focused on HIV and other sexually transmissible infections (67%); followed by comprehensive education and information (10%); contraception counselling/provision (9%); sexual function and psychosexual counselling (5%); fertility care (8%); and gender-based violence prevention, support/care (1%). There were no studies on antenatal/intrapartum/postnatal care and on safe abortion care (0% for both). Conceptually, there was lack of knowledge of the different domains of men’s SRH, with negative attitudes, and many misconceptions; as well as a deficiency of health system policies, strategies and interventions for SRH.

**Conclusion:**

Men’s SRH is not sufficiently prioritized. We observed five ‘paradoxes’: strong focus on HIV/AIDS, when MENA has low prevalence of HIV; weak focus on both fertility and sexual dysfunctions, despite their high prevalence in MENA; no publications on men’s involvement in sexual gender-based violence, despite its frequency across MENA; no studies of men’s involvement in antenatal/intrapartum/postnatal care, despite the international literature valuing such involvement; and, many studies identifying lack of SRH knowledge, but no publications on policies and strategies addressing such shortcoming. These ‘mismatches’ suggest the necessity for efforts to enhance the education of the general population and healthcare workers, as well as improvements across MENA health systems, with future research examining their effects on men’s SRH.

**Supplementary Information:**

The online version contains supplementary material available at 10.1186/s12889-022-14716-2.

## Introduction

Historically, sexual and reproductive health (SRH) and rights were viewed as a woman’s issue [1]. One of the United Nation’s Sustainable Development Goals is ensuring universal access to SRH [2]. However, despite the growing recognition that men also need SRH care, they remain underrepresented in the SRH social debate, care, and research [3]. Interestingly, the impact of male SRH on men’s welfare was only considered as function of women’s SRH rights [4]. Generally, barriers to men’s engagement in SRH include the lack of health insurance, masculinity ideas that conflict with SRH care, stigma related to accessing services, and lack of knowledge regarding available services [5]. For instance, across high- and low-income countries, shortcomings of men’s SRH at the policy level, availability and accessibility of clinical services and acceptability by society are evident [2, 6]. The Middle East and North Africa (MENA) region is no exception.

The Reproductive Health Working Group established in 1988 in Cairo to advance research in the Arab countries and Turkey began with a focus on women [7]. With time, the focus widened to include men’s lives and their impact on women’s health [8]. Three decades later, the situation remains not much changed [9, 10]. Some Arab nations have attempted health system reforms recently, however further efforts are still needed to include SRH [11]. Challenges that appear to hinder the implementation of new strategies that facilitate research of SRH issues among men in MENA include attitudes of male dominancy, and traditional myths that led to stigma and biases when dealing with SRH. In addition, sociopolitical instabilities in MENA have resulted in one of the highest numbers and range of refugees/humanitarian settings globally, associated with collapsed health systems, lack of essential medications and contraceptives, as well as absence of and low access to skilled health care providers (HCP) [12].

Therefore, the current scoping review aimed to outline the knowledge gaps and considerations that impact on men regarding SRH in MENA. Specifically, we appraised the range of factors that bear on men’s SRH in terms of clients/users, healthcare providers (HCPs), healthcare system and sociopolitical environment.

## Methods

### Scoping review

The purpose of a scoping review is not to localize and account for every published information on the topic [13]. Rather, the goal is intentionally wider, to interrogate the literature, discover the important features of the topic, unearth potential gaps, display crucial examples and synthesize research evidence, particularly when the subject has not been meticulously reviewed or is complicated, heterogeneous and assorted [14–16]. Therefore, the scoping review was selected to appraise SRH care for men in MENA. The current scoping review was undertaken in line with The Preferred Reporting Items for Systematic Reviews and Meta-Analyses (PRISMA) extension for scoping reviews [17]. We employed a six-step framework in line with Arksey and O’Malley [18], with procedural and methodological rigor, and clarity/transparency relating to methodology (Table 1) [19–21]. Table 2 outlines the definitions used in this review. The search terms employed are depicted in a supplementary file (Supplementary Box 1). The inclusion criteria employed by the current review included: (1) peer-reviewed empirical studies, all designs were taken into account; (2) published between January 2010 and May 2020; and (3) appraising the experiences of men in SRHC or the healthcare providers’ (HCPs) perspectives on men’s SRHC; and (4) undertaken in the nations of the MENA region. Items that did not meet the inclusion criteria were excluded.

**Table 1:** Six-step framework employed in the present scoping review

**Step**	**Description**
Research questions	What is the current status of the literature published from MENA regarding men and SRH?; How men in MENA experience SRH? These queries were broken into four specific objectives relating to clients/users, HCPs, healthcare system and sociopolitical environment.
Search strategies	Structured literature search using electronic databases that included PubMed and WoS, limited to English language using search terms. (online supplemental Box 1)
Charting the Data	Data extracted consisted of items relevant to specific factors examined. Articles were mapped employing the eight domains of WHO framework for operationalising SRH [19]
Collating, Summarizing, and Reporting Results	Review team assembled, grouped, synthesized and condensed the findings. Theoretical frame work for analysis involved two interrelated schemes [20, 21] (detailed below). Potential gaps were mapped.
Consultation Exercise	Two senior experts specialized in SRH reviewed the findings to provide opinion on and substantiate the findings

Adopted from Arksey and O’Malley [18]

**Table 2:** Terms and definitions used in the current scoping review

**Term**	**Definition**
Sexual health	Integration of the somatic, emotional, intellectual, and social aspects of sexual being, in ways that are positively enriching and that enhance personality, communication, and love [19].
Reproductive health	State of complete physical, mental and social well-being and not merely the absence of disease or infirmity, in all matters relating to the reproductive system and to its functions and processes [2].
MENA	Algeria, Bahrain, Djibouti, Egypt, Iraq, Jordan, Kuwait, Lebanon, Libya, Malta, Morocco, Oman, Qatar, Saudi Arabia, Syria, Tunisia, United Arab Emirates, Palestine, Yemen, Sudan, Western Sahara.
Scoping review	Type of research synthesis that aims to ‘map the literature on a particular topic or research area and provide an opportunity to identify key concepts; gaps in the research; and types and sources of evidence to inform practice, policymaking, and research [16].

*MENA* Middle East and North Africa

The review team systematically synthesized the findings and summarized and presented the charting results as they relate to the review questions and objectives. This included extracting the aims, populations, findings and conclusions of each included study development of an excel sheet. Then groupings were created based on the eight domains of SRH as outlined by WHO [19] framework for operationalising sexual health and its linkages to reproductive health, and further inspired by the findings that were emerging. Any disagreements were resolved by consensus between the team members.

### Theoretical frameworks

The theoretical framework for analysis we employed involved two interrelated schemes. The first is WHO’s (2017) framework for operationalising sexual health and its linkages to reproductive health, comprising 8 domains: antenatal, intrapartum and postnatal care; comprehensive education and information; contraception counselling and provision; gender-based violence prevention, support and care; fertility care; prevention and control of HIV and other sexually transmissible infections (STIs); safe abortion care; sexual function and psychosexual counselling [19].

Then, for each of these domains, we further employed Kilbourne et al’s (2006) framework to outline the health service perspectives on the appreciation of health and healthcare disparities, highlighting the factors influencing men’s experiences, including: individual (HCPs and users); interpersonal (healthcare encounter and contact characteristics); organisational (healthcare system); and the larger influence of the community and public policies (sociopolitical). Our searches of men’s SRH yielded very sparse articles on healthcare encounter and contact characteristics, therefore our domains are organized under 3 categories, namely clients/ HCPs; healthcare system factors; and sociopolitical factors [22].

## Results

### Search results

**Fig. 1:**
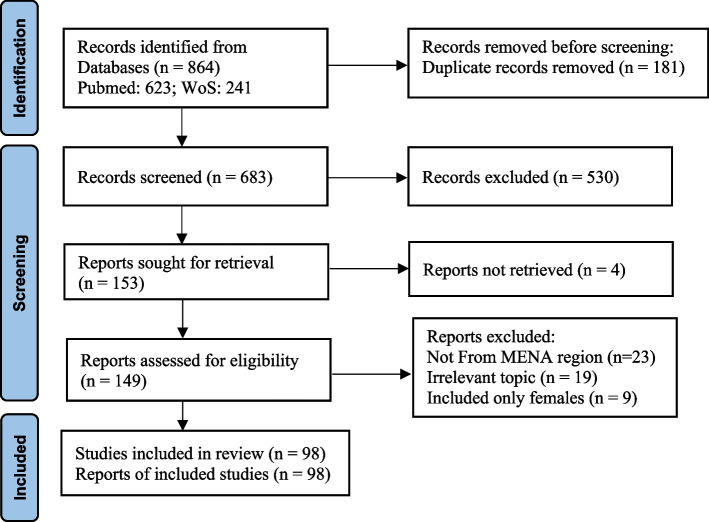
PRISMA flow chart on search results of men’s experiences in sexual and reproductive healthcare in MENA countries [23]

Figure 1 shows the PRISMA flow chart of the search results of men’s experiences in sexual and reproductive healthcare in MENA countries. After searching and screening, a total of 98 articles were finally included in the present review.

**Fig. 2:**
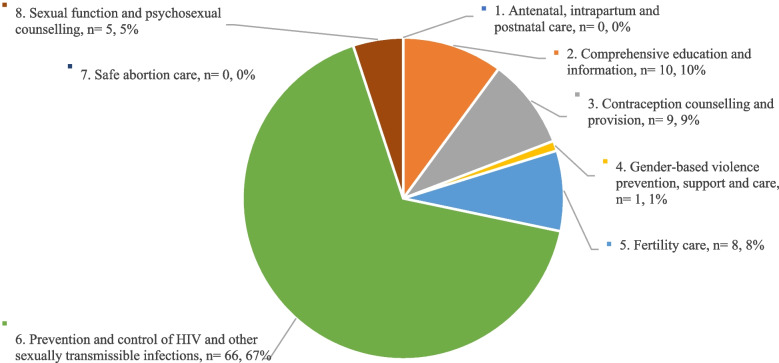
Description of identified studies in terms of WHO’s Framework [19]

Men’s SRH topics were grouped using the WHO framework, illustrating the intertwined features between sexual health and reproductive health [1]. The great majority of studies focused on the prevention and control of HIV and other sexually transmissible infections (67%). Conversely, only 1 study (1%) discussed gender-based violence; and no studies attended to the two domains of antenatal, intrapartum and postnatal care and safe abortion care (0% for both) (Fig. 2).

### Clients and populations

The identified studies included children/adolescents, youth, school/university students, HCPs, particular patient groups, general public or special populations e.g., industrial/tourist workers, refugees, truck drivers, refugees, seafarers, alcohol/drug abusers, men having sex with men and female/male sex workers, and people living with HIV/AIDS (PLWHA) or healthy children/ adolescents of HIV-positive parents (Supplementary Table 1).

### Comprehensive education and information

A total of 10% of the studies we identified were dedicated *per se* to comprehensive education. However, education was generally discussed in the greater majority of the articles that this review identified, focusing on discussions of the knowledge levels of different population groups towards several aspects of SRH in men, exploring the determinants of knowledge gaps among clients and HCPs, as well as health systems and sociopolitical factors (detailed below). Generally, these studies identified a knowledge lack regarding issues relating to men’s SRH in MENA.

### Prevention and control of HIV and other sexually transmissible infections

Most studies of the review assessed the knowledge of a range of population groups pertaining to prevention and control of STIs and HIV/AIDS. More focus was directed towards knowledge level among clients/ users than of HCPs. Overall, there was low knowledge level across most of the groups that were assessed. Knowledge level was influenced by factors including age, sex, level of education and experience with age. Knowledge deficits often translated into negative attitudes towards HIV/AIDS or PLWHA. In addition, little is known about the perception of the magnitude of HIV/AIDS in MENA (Table 3).

**Table 3:** HIV and STIs in MENA: characteristics of users and health care professionals

Characteristic	Clients/ users	Health Care Professionals
Knowledge	▸ School/ High school students: HIV/AIDS knowledge and prevention: inadequate; better among high school students; needed more understanding to prevent stigmatization/ discrimination of infected persons, knowledge varied significantly by country and gender [24–27]. Most boys knew about AIDS but rarely other STIs [28]. ▸ University students • HIV/AIDS: medical university students were aware of HIV, its transmission and prevention [29], with few misconceptions [30]. Conversely, dental students had low to moderate knowledge with high misconceptions and paramedical students had low knowledge [31, 32], knowledge was sometimes associated with being male and higher years of study [32, 33]. • STIs: male medical students and dental students had low HPV knowledge [34]; clinical-years associated with better knowledge [35]. ▸ General population • HIV/AIDS: deficient knowledge, with misconceptions about prevention [36, 37]. Knowledge was positively associated with education, age, residence, experience, and socioeconomic status [38, 39]. • STIs: low HPV awareness, which was better among older clients and females [40]. ▸ Special populations: HIV knowledge was high among PLWHA and alcohol/ drug abusers especially men with high education [41, 42], satisfactory in seafarers, but with some misconceptions, and low in refugees and dental patients [43–45].	▸ Physicians: PHC physicians sometimes had never managed an AIDS case; had low HIV/AIDS transmission, treatment and risk behaviour knowledge [46]. Knowledge was associated with years of experience, status/specialty and practice location [47]. ▸ Dentists: moderate knowledge about oral HIV manifestations and transmission [48]. ▸ Nurses: low HIV/AIDS disease and prevention, however, had high knowledge in risk groups identification [49].
Attitude	▸ School/ high school students: negative attitudes toward AIDS and PLWHA but were willing to be HIV tested [31]. ▸ University students: undergraduates displayed moderate acceptance of PLWHA, and most were willing to care for an HIV-infected person, although attitudes fluctuated between equivocal or negative which was related to lack of HIV knowledge [29, 30, 50]. HPV vaccination was acceptable by male medical students and dental students [34], more among clinical-year students, those vaccinated for hepatitis B, and with higher HPV knowledge [35]. ▸ General population: although individuals expressed eagerness to know more about HIV/AIDS [51], a sense of fatalism regarding HIV acquisition was common [36], with negative attitude toward PLWHA. Factors affecting attitude were age, sex, marital or social status, educational level, experience, and nationality [52]. ▸ Special populations • PLWHA: low adherence to treatment [53]. • Seafarers, sex workers and refugees: high risk behaviors [43, 44, 54]. • Most alcohol/ drug abusers: negative attitudes towards PLWHA, but 55.5% felt sympathy for them [42].	▸ Physicians: most PHC physicians suggested isolating PLWHA in isolated places/hospitals [46]. ▸ Nurses: negative attitudes toward PLWHA/ suspected HIV cases (injecting drug users, MSM, sex workers), refusing to provide care or get blood sample; most reported that HIV patients should be ashamed of themselves [49, 55]. Attitude barriers to care included fear of getting infected with HIV, disbelief in effectiveness of infection control measures, misconceptions, fear of stigmatization, and moral judgments [56].
Perceptions	▸ Kuwait: majority of participants were satisfied with the government’s policy for AIDS prevention; and proposed that religion is important in dealing with HIV infection [38]. ▸ Egypt: compared to industrial workers, tourism workers had a better perception of the magnitude of the HIV/AIDS problem worldwide and in Egypt, and the likelihood of it worsening [57].	

*HCPs* Health care professionals, *PHC* Primary healthcare, *HPV* Human papilloma virus, *PLWHA* People living with HIV/AIDS, *MSM* Men who have sex with men, *STIs* Sexually transmitted infections

In terms of the healthcare system, for HIV, much had been achieved in linking to and retention in care, antiretroviral therapy coverage and viral suppression, despite obstacles in prevention programs e.g., deficient funding and infection control due to lack in supplies and procedures as well as insufficient data/surveillance [58, 59]. The financial burden/ healthcare costs of PLWHA varied, depending on the presenting illness, clinical stage, developed opportunistic infection, co-morbidity, and pharmacological therapy [60], with empirical results illustrating a negative relationship between both public and private healthcare spending and HIV [61]. Policies and protocols regarding dealing with PLWHA were also absent [55]. In addition, war had restricted the surveillance activities e.g., access to voluntary counseling and treatment (VCT) centers in Syria [62]. Therefore, many opportunities for HIV testing, based on at-risk behaviors or clinical signs, were missed [63].

Several HIV awareness programs have been implemented. At hospital level, introduction of multi-disciplinary team (MDT) approach in managing HIV patients resulted in statistically significant control of the disease [64]. On the population level, school-based HIV education interventions exhibited mixed effectiveness in improving knowledge of HIV transmission/ prevention [24, 65, 66]. The key enabling factors were high quality of training for peer educators, supportive school principals, and parental acceptance of the intervention [67]. Community prevention in Sudan and Yemen was directed to the general public as well as to men who have sex with men (MSM) and female sex worker (FSW), focusing on behavioural change, enabling supportive environments and providing support for PLWHA [58]. Likewise, community-based educational interventions targeting truck drivers was effective in increasing coverage of HIV testing and counseling [68]. NGOs In Lebanon adopted HIV self-testing but uptake was low due to noncompliance of beneficiaries and lack of human/financial resources. Interestingly, self-testing was much improved during COVID-19 because of the absence of on-site activities, shifting more efforts towards HIV self-testing [69]. Generally, the apparent trend that this review observed was a deficiency of studies addressing the healthcare system and policies pertaining to the prevention and control of HIV and other STIs.

As for the social factors, high stigma and discrimination toward PLWHA were present [26, 39], rooted in values and fears, and manifesting in reluctance to use the same health facilities as PLWHA [70]. PLWHA faced such stigma in their homes and at work, forcing them to seek support from NGOs or close family. This stigma affected their disclosure to the wider community due to uncertainty of the repercussions, leading to a lonely life and financial difficulties [41]. In addition, HIV testing uptake was limited by concerns about confidentiality and fear of repercussions on health and employment [71]. Stigmatization of PLWHA was inversely related to HIV/AIDS knowledge [47, 72]. Stigma extended to physicians providing care for PLWHA, caused by fear of infection, to the extent of community unwillingness to use those physicians’ services. On the other hand, stigma toward physicians who refused to provide care was linked to perceptions of unethical behavior [70]. Victimization was also evident, e.g., most Saudi students believed that PLWHA were responsible for their infection and that AIDS was a Godly punishment [72]. Collectively, the apparent trend we observed in terms of the studies in this review was the generalized stigmatization of the PLWHA as well as in some cases the HCWs dealing with them.

### Fertility care/ sexual function and psychosexual counselling

Some end-users displayed limited/inadequate knowledge about the concept, availability and benefits of SRH, voiced by the need for more information and quality services [73]. Healthcare workers sometimes exhibited deficient knowledge with regards to male SRH services [73–76]. This led to different personal attitudes towards the problem that was affected by age, sex and level of education [77] (Table 4). As for sexual health, although the knowledge level was acceptable, however, the sociopolitical norms affected the proper attitude towards the topic. This goes for general population as well as HCP (Table 4).

**Table 4:** Fertility care, sexual function and psychosexual counselling in MENA: characteristics of users and health care professionals

Characteristic	Clients/users	Health Care Professionals
Knowledge	▸ RH: inadequate knowledge about the concept, availability and benefits [73]. ▸ Premarital checkup: • Egypt: lack of knowledge among general population even among educated respondents [78]. • KSA: university students aware of its importance in preventing transmission of hereditary diseases to offsprings and ensuring their partner’s health [79]. ▸ Sexual Health: • Younger boys: more aware of physiological/emotional puberty changes of their own sex; but not of opposite sex [28]. • Adults: dialogue between patients and their treating physicians regarding ED assists patients to seek proper/safe medical advice [80].	▸ Healthcare workers sometimes displayed low knowledge e.g., about ICSI [73, 75, 76]. ▸ Clinical practitioners: more likely to have accurate knowledge of FP options than oncologists [74]. ▸ Sexual Health: • Urologists: more knowledgeable about ED, but gynecologists had better attitude towards ED patients [81]. • Nurses: most were not very knowledgeable about/confident to address sexuality, viewing it as not within their responsibilities [82, 83]. • HCP: lacked confidence in their sex education skills and knowledge [84].
Attitude	▸ RH services: users not always satisfied with HCP attitudes, stating it was unpleasant, with poor communication and inappropriate management approach [73]. ▸ Premarital checkup: • Egypt: among general population, most respondents, except unmarried males, had favorable attitude [78]. • KSA: most university students had generally positive attitude and good intended practices toward PMS. Most participants demanded implementing a law that prohibits incompatible marriages [79]. ▸ Sexual Health: • Many adolescent boys found female genital cutting necessary, favoured polygamous marriage at younger age, but not consanguineous marriages [28]. • ED: sensitive issue among older clients, hence in rarely consulting. Conversely, university students were more liberal toward sex, had more risky behaviours [80, 85]. • Gay communities: highly knowledgeable but had high-risk behavior (low condom use/ HIV testing), most disclosed their sexual orientation only to their partners and not to their HCP even if needed [86].	▸ Physicians and HCP with previous SRH had better youth-friendly attitudes [87]. ▸ Family physicians: favorable attitudes toward infertility management, but attitude varied with age, gender and experience [77]. ▸ Oncologists: low perception of importance of FP, leading to poor referral to specialists; gender bias in informing males about FP options prior to cancer treatment compared to females [74, 75]. ▸ Sexual Health: • Nurses: negative attitude influenced by their beliefs about sex/sex education (viewing early sex education as problematic), negative attitude was associated with sex (female) and no previous training on sexuality [82–84]. • Lebanon: HCP had positive attitudes towards LGBT patients; mental health providers less likely to believe that homosexuality is mental health disorder, but more likely a natural variation on the sexual orientation spectrum [88].

*HCP* Health care professionals, *KSA* Kingdom of Saudi Arabia, *PMS* Pre-marital screening, *ED* Erectile dysfunction, *LGBT* Lesbian, gay, bisexual, and transgender, *FP* Fertility preservation, *SRH* Sexual and reproductive health, *RH* Reproductive health, *ICSI* Intracytoplasmic sperm injection

Healthcare system-related factors suggested that improvements in quality of infertility management required evidence-based training, supplies, laboratory/radiology support, improved communications with specialists, and availability of guidelines [77]. Youth also felt that SRH services needed to be easily accessible and have equal geographical distribution [73]. Reproductive tourism attracted patients from countries with deficient invitro fertilization (IVF) services or policy restrictions, and required high-tech medical settings, with visa regulations allowing users to complete an entire IVF cycle [89].

### Contraception counselling and provision

The knowledge and acceptance of family planning varied across MENA. Generally, good awareness of contraceptive approaches was mainly for women’s methods but not for male contraception. However, such awareness was not translated into increased application/use of family planning in many MENA countries due to religious and sociocultural norms surrounding this topic. The lack of knowledge about male contraception was also including HCP e.g., pharmacists (Table 5).

**Table 5:** Contraception counselling and provision in MENA: characteristics of users and health care professionals

Characteristic	Clients/users	Health Care Professionals
Knowledge	• UAE: most men aware of availability of male contraceptive methods, only few currently used them, mainly condoms, and only 1.1% were sterilized. Few thought that contraceptive pills/ monthly injection for men are available [90]. • Jordan: most men heard about family planning, most commonly intrauterine device and oral contraceptives [91]. • Iraq: decreased knowledge regarding correct condom use and its effectiveness for contraception/ STIs prevention [92]. • Egypt: most secondary-school pupils knew about contraception, girls had more information [93].	Pharmacists: decreased knowledge about male OCPs and their mechanism of action, with negative perceptions towards them. Barriers to male OCPs were cultural norms, side effects, and poor compliance [94].
Attitude	• UAE: majority of men rejected male contraception, due to religious reasons, followed by cultural barriers, personal beliefs, medical disorders and economic factors. Male contraception use significantly associated with high education level of partners, low family size and family income [90]. • Jordan: married men had good attitudes/knowledge about family planning, but only 45.1% currently used contraception. However, most men agreed about a minimum 2 years’ child spacing and starting contraception after childbirth and that husband and wife should share decisions about family planning and number of children [91]. • Sudan: three-fifths of men with reproductive age wives wished to use family planning services but only about one-fifth currently used an effective method. Men were more interested in learning more about female than male sterilization [95]. • Iraq: condoms were rarely used for family planning due to lack of need, fertility-related reasons or use of female contraception methods [92]. • Egypt: secondary-school pupils agreed about using contraceptive methods in the future [93].	

*HCP* Health care professionals, *UAE* United Arab Emirates, *STIs* Sexually transmitted infections, *OCPs* Oral contraceptive pills

### Sex-based violence

Little information exists on gender-based violence in MENA, and our search yielded only 1 article. In Egypt, the majority of street children experienced more than one risk including harassment or abuse by police and other street children, drug abuse, and, among sexually active 15–17-year-olds, most reported multiple partners and never using condoms, and most girls had experienced sexual abuse [96]. Such behaviors put them in substantial overlap with populations at highest risk for HIV, namely men who have sex with men, commercial sex workers, and injection drug users [96].

## Discussion

Addressing SRH of men alongside that of women’s is essential. However, it has not received the attention it deserves worldwide. We outlined the current knowledge, knowledge gaps and considerations that impact on men’s SRH in MENA, and appraised the HCPs’, users’, healthcare systems’ and social factors affecting such services. To our knowledge, this is the first comprehensive scoping review of men’s SRH in MENA.

Our main findings unearthed a strong HIV/AIDS focus of the published outputs, but a much weaker focus on issues related to fertility care, sexual dysfunctions/ counselling, and gender-based violence. The general population, different clientele groups, and a range of HCPs exhibited many SRH knowledge gaps, that subsequently lead to a high prevalence of unfavorable attitudes towards men’s SRH conditions, stigmatization, and the emergence of many misconceptions. Generally, across the range of countries under examination, the quality of SRH services could be improved. Surprisingly, we could not find published data on legislation, government policies or national SRH strategies. More importantly, we observed several paradoxes in terms of the lack of congruence between many of the domains that the published outputs addressed on the one hand; and the actual ‘on the ground’ situation across MENA on the other.

The first paradox pertained to the strong HIV/AIDS focus across the published literature, despite the low prevalence/ burden in MENA region (0.1%) [97, 98]. While it is difficult to speculate the reasons behind such discrepancy, perhaps it might be explained by the wide international interest and availability of funding to explore epidemiological and behavioural HIV/AIDS research, as evidenced by that most studies were funded by multi-lateral bodies e.g., UNICEF or philanthropic agencies [56, 67, 99]. Notwithstanding, war and political instabilities may increase the vulnerability of the region to HIV by reducing access to prevention services, destroying health care infrastructure, disrupting social support networks, increasing exposure to sexual violence, and expanding immigration and displacement [100]. Despite the tremendous efforts made in the global cognition and epidemiology of HIV infection, knowledge in MENA remains limited and controversial [99]. The large number of published papers on HIV/AIDS we observed concurs with the findings of a scoping review of men’s SRH in Nordic countries, where out of 68 studies that were identified, 15 papers dealt with STIs, mainly HIV (12 papers) and MSM (9 papers) [101].

The second paradox was the weak focus of the published articles on the two topics of fertility care and sexual dysfunctions/counselling, despite the high prevalence of fertility and sexual problems in MENA (22.6%) [102]. Such misfit might by due to the complex cultural, religious, community gender and social norms prevalent among MENA populations that render them reluctant to disclose their SRH concerns [80]. Likewise, MENA has low knowledge of sexual relationships, attributed to a lack of sex education in schools and the conservative culture of the community, factors that might contribute to the increasing prevalence of e.g., premature ejaculation in the region [103]. Similarly, erectile dysfunction (ED) is quite prevalent among Arab men, probably explained by the high prevalence of endothelial dysfunction risk [104]. Again, our findings support a review that scoped men’s SRH across Scandinavia, where sexual functioning/ counselling studies covered a very small proportion (2/68) of the studies that the review identified [101].

The third paradox we observed was related to gender-based violence. The current review found that publications of gender-based violence prevention, support and care represented only 2% of the retrieved studies. This is despite that women’s exposure to male domination has long been normalized in the Arab world [105, 106]. This could be due to possible gender disparity and male predominance in MENA representing barriers to such research [107]. Elsewhere, female researchers lag behind their male counterparts in successfully receiving grants [108]. Even though the prevalence of intimate partner violence (IPV) is high across Arab countries, evidence on its correlates remains limited [109]. The situation is complicated by the fact that the West’s social acceptance of divorce is not shared by Arab nations, where the centrality of marriage and family culture persists, and divorce continues to be stigmatized [109]. A scoping review of men’s SRH in the Nordic countries found that published studies about sexual violence comprised a very small minority (2/68) of the studies [101], concurring with our findings. Hence, efforts to mitigate gender gap and promote equity, diversity, and inclusion of females in research may improve any gender-based parity in research topics.

The fourth paradox was that the present review found no studies pertaining to men’s SRH that addressed the domain of antenatal, intrapartum and postnatal care (0%), despite that men’s involvement in maternal health programs is a key to increase utilization of maternal health services [110]. This could be due to prohibition of gender mixing in antenatal, intrapartum and postnatal care as well as in women’s hospital settings, hence disallowing male presence in such encounters. Such lack supports that in spite of the growing recognition of father’s importance for early family health/well-being, there has been very limited attention to men’s own experiences and developmental needs during their partner’s antenatal visits [111, 112]. Nevertheless, our observations contrasts with the Nordic study, where more than one-third of the papers were related to experiences of expectant fathers during antenatal, intrapartum and postnatal care [101]. Empowering men with antenatal care knowledge and joint decision-making with their spouses increases male involvement [113], particularly that complex community sociocultural norms and social stigma are barriers to men’s attendance at antenatal care services with their partners [114]. The present maternity health policies in Arab countries might need revision to allow fathers’ inclusion [115]; and our findings suggest a need for communication, education, and information-based health promotion programs that empower men in these domains.

The fifth paradox was that a great proportion of studies discussed knowledge levels and gaps pertaining to men’s SRH in MENA, identifying a range of factors that influence knowledge. Surprisingly, there was no parallel body of literature debating effective interventions and their implementation in order to remedy/overcome such gaps. Similarly, we found no articles dedicated to policies and strategies to address such shortcomings at state, health care system and public health policy making level, when certainly improving men’s access to SRH requires state, health care system and health care providers interventions and policies [4, 101].

Likewise, the present review found no studies of men’s SRH that addressed safe abortion care (0%), not surprising given that abortion is illegal across most of the region, and there are various legal and societal barriers to the practice in the countries where it is allowed [116]. Very few publications exist on abortion in MENA, and those that do exist tend to give an overview of the legislation of different states or evaluate Islam’s position on abortion [117–121]. Detailed studies on actual medical practices, political debates, local legal implementation, moral/social norms, and trajectories of MENA women are very rare [122–126], let alone those on men’s participation in safe abortion care.

A unique characteristic across MENA is the prevalent political instabilities and refugee situations [44]. MENA has a current 16 million forcibly displaced and stateless people [127], situations that increase risky behavior for HIV and STIs [44]. Our findings resonate with Uganda, where refugee adolescents and displaced youth were a key population left behind in HIV prevention efforts [128]. Young refugees have limited access to sexual health information and resources in their resettlement places, highlighting a need for sexual health education programs for men and women as part of resettlement services [129]. Notwithstanding, Egypt has taken welcome steps: policies in progress include commitment to give refugees access to primary health care and education within national systems, and refugees are currently covered by the universal health care insurance scheme on equal footing with Egyptians [130].

The current scoping review has limitations. Surveys and reports about MENA health issues are mainly published in local languages and hardly accessible through electronic databases [102]. The study has many strengths. To our knowledge, this study is the first scoping review focusing on men’s experiences in SRH care across MENA. There was no time limitation for our literature search. In line with others [14, 15], the review was driven by a strict peer reviewed protocol. For an appropriate search, we examined the search strategy used in a similar published article on men’s SRH in Nordic countries [101] and modified the search terms they used. We searched four electronic bibliographic databases and reference lists of articles. Despite that our search was conducted using only English terms, our review included any articles published in English or Arabic; given that Arabic is the major language in MENA. The screening and data characterization forms that were employed were pretested by all members of the reviewer team and modified as appropriate before the review. Three training sessions included the completion of the screening and data characterization forms, using articles that were randomly selected from the literature. Data extraction of each article was undertaken by two independent members of the review team (WEA, MA) who worked simultaneously together on each article, and any disagreements were resolved by discussion. The review team systematically synthesized the findings by extracting the aims, populations, findings and conclusions of each included study and categorizing them based on the eight WHO SRH domains.

## Conclusion

It is important for men to access SRH care. The available literature from the MENA region suggests that men’s SRH is not sufficiently prioritized. A detailed landscape of men’s experiences in SRH care across these countries remains to be explored. A number of pertinent ‘mismatches’ were evident in the literature. There was strong focus on HIV/AIDS, when MENA has the lowest prevalence of HIV in the world; a much weaker focus on fertility and sexual dysfunctions, despite that these conditions were much more prevalent in MENA countries; no publications on men’s involvement in sexual gender-based violence, despite women’s exposure to its various forms across the MENA region; and no studies pertaining to men’s SRH in terms of their involvement in antenatal, intrapartum and postnatal care, despite the prevailing international literature to the value of such involvement. Such incongruencies might serve to provide future direction for the formulation and implementation of comprehensive strategies to help tackle men’s SRH challenges in MENA region. These could include strengthening the current policies, strategies and interventions to enhance and improve the attitudes, behaviors and education of the general public, the youth, men in general as well as HCPs. Furthermore, strengthening the health systems over time in terms of political commitment, structures, organization, funding and interventions to address core issues and to more formally respond to men’s SRH challenges in MENA would be welcomed. Future research should examine the influence of policies and the healthcare service delivery and organization on men’s access and experiences in SRH care.

## Supplementary Information

**Additional file 1: Supplementary Box 1.** Search terms used in the current scoping review. **Supplementary Table 1.** Included articles on men's experiences of sexual and reproductive healthcare in MENA countries [131–154].

## Data Availability

The datasets used and/or analysed during the current study are available from the corresponding author on reasonable request.
