# Supplementary material for: Scoping review of sexual and reproductive healthcare for men in the MENA (Middle East and North Africa) region: a handful of paradoxes?
Source: BMC Public Health. 2023 Mar 27;23:564. doi: 10.1186/s12889-022-14716-2 (PMC10040932; doi:10.1186/s12889-022-14716-2)
Supplement: Supplementary file 1 — Additional file 1: Supplementary Box 1. Search terms used in the current scoping review. Supplementary Table 1. Included articles on men's experiences of sexual and reproductive healthcare in MENA countries [131–154]. [file 12889_2022_14716_MOESM1_ESM.docx]

**Supplementary Box 1: Search terms used in the current scoping review**

| (Sexual Health[mesh] OR "Sexual health" OR Reproductive Health[mesh] OR "Reproductive health" OR "Sexual and reproductive health" OR "Sexual and reproductive health and rights" OR SRHR OR "sexual function" OR "sexual functions" OR "sexual dysfunction" OR "sexual dysfunctions" OR erectile dysfunction[mesh] OR "erectile dysfunction" OR sexual satisfaction OR sex offenses[mesh] OR sexual violence OR gender-based violence[mesh] OR gender-based violence OR family planning services[mesh] OR "Family Planning" OR contraceptive agents[mesh] OR contraceptive devices[mesh] OR "contraceptive" OR "contraceptives" OR Condom[mesh] OR condom OR condoms OR Infertility[mesh] OR infertility OR fertility[mesh] OR fertility OR prostatic neoplasms[mesh] OR "Prostate cancer" OR Genital Diseases, Male[mesh] OR sexually transmitted diseases[mesh] OR "Sexually transmitted infections" OR STIs OR "Sexually transmitted diseases" OR STDs OR chlamydia[mesh] OR Chlamydia OR Gonorrhoea[mesh] OR gonorrhea OR gonorrhoeae OR Syphilis[mesh] OR Syphilis OR Trichomonas Infections[mesh] OR Trichomoniasis OR Herpes Genitalis[mesh] OR "herpes genitalis"[tiab] OR Papillomavirus Infections[mesh] OR "Papillomavirus" OR Condylomata Acuminata[mesh] OR "Genital warts" OR HIV Infections[mesh]) AND (men[mesh] OR Men OR man OR men’s OR Male[mesh] OR male OR masculinity[mesh] OR masculinity OR Men's Health[mesh] OR Homosexuality, Male[mesh] OR "MSM" OR "men having sex with men"[TIAB] OR "men who have sex with men"[TIAB] OR "men who have sex with other men"[TIAB] OR ((transgender*[TIAB] OR transgender persons[MH] OR transsexual*[TIAB]) AND man) OR transman[TIAB] OR "trans men"[TIAB] OR transmen[TIAB]) AND (Health Services Accessibility[mesh] OR Health Facilities[mesh] OR community health services[mesh] OR health services[mesh] OR health services research[mesh] OR delivery of health care[mesh] OR preventive health services[mesh] OR health services needs and demand[MH] OR quality of health care[majr:noexp] OR "Health care providers" OR "Health care"[tiab] OR "Health services"[tiab] OR "Healthcare"[tiab] OR "healthcare"[tiab] OR clinic[tiab] OR hospital[tiab] OR "primary care"[tiab]) AND (Professional-Patient Relations[mesh] OR Attitude of Health Personnel[mesh] OR Patient Satisfaction[mesh] OR Healthcare Disparities[mesh] OR patient acceptance of health care[MH] OR health care evaluation mechanisms[mesh] OR "perception"[tiab] OR "perceptions"[tiab] OR "perceive"[tiab] OR "perceived"[tiab] OR "satisfaction"[tiab] OR "expectation"[tiab] OR "expectations"[tiab] OR "experience"[tiab] OR "experiences"[tiab] OR "evaluation"[tiab] OR "assessment"[tiab] OR "quality"[tiab] OR "trust"[tiab] OR "Shame"[tiab] OR "stigma"[tiab]) AND (Epidemiologic Research Design[mesh] OR Qualitative Research[mesh] OR empirical research[mesh] OR Surveys and Questionnaires[mesh] OR survey OR surveys OR questionnaires OR questionnaire OR Empirical OR Cross-Sectional Studies OR Cohort OR CaseControl OR Observational OR Registries OR analysis OR Clinical Trials OR Meta-Analysis OR "meta analysis" OR "systematic review" OR "scoping review" OR "literature review" OR "review of literature" OR Qualitative OR "Grounded Theory" OR Interviews as Topic[mesh] OR "interviews" OR "interview" OR focus groups[mesh] OR "Focus group" OR "focus groups" OR themes[tiab]) AND (Arabic Countries[mesh] OR arabian[tiab] OR Egypt[tiab] OR Egyptian[tiab] OR Libya[tiab] OR Libyan[tiab] OR Tunis[tiab] OR Tunisian[tiab] OR Algeria[tiab] OR Algerian[tiab] OR Morocco[tiab] OR Moroccan[tiab] OR Sudan[tiab]OR Sudanese[tiab] OR Jordan[tiab] OR Jordanian[tiab] OR Iraq[tiab] OR Iraqi[tiab] OR Lebanon[tiab] OR lebanese[tiab] OR Saudi Arabia[tiab] OR Saudi Arabian[tiab] OR kuwait[tiab] OR Kuwaiti[tiab] OR United Arab Emirates[tiab] OR UAE[tiab] OR U.A.E.[tiab] OR djibouti[tiab] OR djiboutian[tiab] OR Syria[tiab] OR Syrian[tiab] OR muritania[tiab] OR muritanian[tiab] OR Yemen[tiab] OR Yemeni[tiab] OR Palestine[tiab] OR Palestinian[tiab]) |
| --- |

***Supplementary Table 1*: Included articles on men's experiences of sexual and reproductive healthcare in MENA countries**

| **Author/ year** | **SRH D** | **Country** | **Aim** | **Population/ Concepts discussed** |
| --- | --- | --- | --- | --- |
| Mustafa 1984^94^ | 3 | Sudan | KA towards and use of contraception among men | Male: KA towards family planning |
| Stycos 1985^130^ | 3 *^a^* | Egypt | Assess improvements in measurement of variables, sampling, analytic techniques | General population: KA towards family planning. Healthcare system: quality and programs |
| Eshra 1989^77^ | 2 | Egypt | KA toward premarital counselling/examination | General population: KA towards premarital counselling |
| Abolfotouh 1995^23^ | 6 | KSA | Secondary school students AIDS KAB; impact of AIDS education lecture | Male school-age adolescents: Knowledge about HIV |
| Mahfouz 1995^45^ | 6 | KSA | KA towards AIDS among practicing physicians | Primary Healthcare physicians: KA towards HIV/AIDS |
| Sallam 1995^131^ | 6 | Egypt | Assess continuing medical education (CME) needs regarding AIDS | Physicians Knowledge towards HIV/AIDS |
| Salama 1998^41^ | 6 | Egypt | HIV/AIDS KAP of alcohol/drug abusers; effect of health education on KA | Alcohol and drug abusers: KA towards HIV/AIDS |
| Al-Owaish 1999^37^ | 6 | Kuwait | KABP regarding HIV/AIDS | General population: KA towards HIV/AIDS |
| Saleh 1999^65^ | 6 | KSA | AIDS knowledge in secondary school students; effect of health education on knowledge, transmission; misperception about transmission via casual contact | Students of secondary schools: Knowledge towards HIV/AIDS |
| Warren 1999^132^ | 3 | Jordan | Family planning and reproductive health attitudes of husbands in Jordan | Men: KA towards family planning |
| Al-Serouri 2002^38^ | 6 | Yemen | AIDS causes, transmission, treatment, prognosis; attitudes towards AIDS/ AIDS patients; source of AIDS information; assess risk perceptions | General population: KA towards HIV/AIDS |
| Fido 2002^133^ | 6 | Kuwait | Kuwaiti family physicians AE about HIV/AIDS | Family physicians: KA towards HIV/AIDS |
| Ghazal-Aswad 2002^89^ | 3 | UAE | KP of contraception among UAE men | Men: Knowledge and practice of contraception |
| Shama 2002^50^ | 6 | Egypt | HIV/AIDS perceptions/ risky behaviors of adults in squatter areas; relevant messages/health education that can be used to prevent HIV transmission | General population: KA towards HIV/AIDS |
| Al-Jabri 2003^28^ | 6 | Oman | KAB of medical/ non-medical University students toward AIDS | Undergraduate medical and non-medical students:Healthcare system: Quality and Programs |
| Holt 2003^43^ | 6 | Sudan | KAb, HIV/STI prevalence of Sudanese refugees and Ethiopian sex workers | Refugees: KA towards HIV/AIDS |
| Abdulmohsen 2004^80^ | 8 *^b^* | KSA | Physician KAP towards ED | Physicians: Knowledge about erectile dysfunction |
| Al-Ghanim 2005^36^ | 6 *^b^* | KSA | General public KA towards HIV/AIDS | General population: KA towards HIV/AIDS |
| Al-Mazrou 2005^30^ | 6 | KSA | HIV/AIDS KA of Saudi paramedical students | Paramedical student: KA towards HIV/AIDS |
| El-Sony 2006^134^ | 6 | Sudan | Cost of managing HIV-positive/negative TB patients | Healthcare system: Cost of programs for HIV |
| Jaffer 2006^27^ | 2 *^c^* | Egypt | KA of Omani adolescents towards reproductive health and factors accounting for them | Secondary-school pupils: KA towards reproductive health |
| Gańczak 2007^49^ | 6 | UAE | KA and educational needs of young people | University students: KA towards HIV/AIDS |
| El-Sayyed 2008^56^ | 6 | Egypt | KAP towards HIV/AIDS infection among Egyptian industry/ tourism workers | Industrial and tourist workers: KA towards HIV/AIDS |
| Kobeissi 2008^135^ | 5 | Lebanon | Long-term impacts of Lebanese civil war on male infertility | Men: Victimization of Male Fertility |
| Nasir 2008^31^ | 6 | Sudan | Prevalence/ socio-economic correlates of dental students' knowledge, information sources and need for further HIV/AIDS education | Dental students: KA towards HIV/AIDS |
| Abdulah Al Turki 2009^79^ | 8 *^b^* | KSA | Cardiovascular risk factors among men with ED at hospital-based PCC | Men: Attitudes towards ED and cardiovascular disease |
| Al-Iryani 2009^25^ | 6 | Yemen | Knowledge of HIV transmission/ prevention; stigma/ discrimination of students toward PLWH | Adolescents: KA towards HIV/AIDS |
| Kahhaleh 2009^136^ | 6 | Lebanon | Impact of HIV prevention interventions in Lebanon since 1996 | General population: KA towards HIV/AIDS |
| Al-Iryani 2010^98^ | 6 | Yemen | Factors that facilitate community peer education for youth HIV prevention | Young people: KA towards HIV/AIDS |
| Al-Serouri 2010^137^ | 6 | Yemen | Young people knowledge about HIV/AIDS transmission, attitudes towards PLWHA, and self-protection from infection | General population: KA towards HIV/AIDS |
| Badahdah 2010^71^ | 6 | KSA | Contribution of shame as cultural value to stigmatization of PLWHA | General population: Knowledge, stigmatization and victimization to HIV/AIDS |
| Kabbash 2010^92^ | 6 | Egypt | Client profile and satisfaction to identify strengths/weaknesses in the service | General population: attitude towards HIV/AIDS screening |
| Kabbash 2010^138^ | 6 | Egypt | HCP satisfaction at VCTs to identify strength/s weaknesses in the service | HCP: attitude and satisfaction towards HIV/AIDS screening |
| Inhorn 2010^88^ | 5 | UAE | Reproductive tourism to/from UAE; individual/population movements for reproductive/other medical care | General population: availability and accessibility of ART services |
| Khalaf 2010^72^ | 2 | Jordan | Needs for RHS among youth; identifying problems when using RHS; identifying youths’ perceptions of the characteristics of youth-friendly RHS | Youth: Knowledge and attitude towards SRH services |
| Nada 2010^95^ | 4 | Egypt | Gender based violence and HIV/AIDS risk behaviors of boys/girls aged 12–17 years living on the streets | Children: Gender based violence, and knowledge and attitude towards HIV/AIDS |
| Rabah 2010^75^ | 5 | KSA | Awareness/views about cryopreservation among oncologists | Oncologists: KA towards fertility preservation |
| Al-Iryani 2011^64^ | 6 | Yemen | School-based peer education for HIV prevention among students | Adolescents: KA towards HIV/AIDS |
| Hassan 2011^48^ | 6 | Jordan | Nurses KA toward HIV/AIDS patients, sources of information and education | Nurses: KA towards HIV/AIDS |
| Arafa 2011^74^ | 5 | Egypt | **E**xplore current oncologists’ practice recommendations, attitudes, and knowledge about fertility preservation in men and women of reproductive age | Oncologists: KA towards fertility preservation |
| Ellepola 2011^139^ | 6 | Kuwait | HIV/AIDS KA amongst Kuwait university dental students | Dental students: KA towards HIV/AIDS |
| Al-Rabeei 2012^29^ | 6 | Yemen | KAB towards HIV/AIDS among students at health institutes | Students of health institutes: KA towards HIV/AIDS |
| Boneberger 2012^26^ | 6 | MENA | School adolescents’ HIV/AIDS knowledge, 7 MENA countries | Adolescents: Knowledge and attitude towards HIV/AIDS |
| Ismael 2012^91^ | 6 *^d^* | Iraq | KAP + socio-demographic factors of condom use among males (15-49 years) | Male: KA towards family planning |
| Eldein 2013^76^ | 5 | Egypt | Family physician attitude/practice of infertility management at primary care | Family physicians: Attitude and training for infertility assessment and fertility healthcare system availability |
| Al-Iryani 2013^66^ | 6 | Yemen | Evaluation in high schools of facilitating/ inhibiting factors for peer education | Adolescents: KA towards HIV/AIDS |
| Valadez 2013^53^ | 6 | Libya | HIV infection level and socio-demographic and behavioural characteristics of MSM and FSW to inform design/future evaluation of National HIV Strategy | MSM and FSW: Healthcare system, quality and programs |
| Nasir 2013^44^ | 6 | Sudan | Associations of HIV-related knowledge/fear of contagion in dental environments and OIDP among patients; if associations modified by frequency of dental service | Dental patients: KA towards HIV/AIDS |
| Alkhasawneh 2014^35^ | 6 *^b^* | Oman | HIV KAB | General population: KA towards HIV/AIDS |
| Farrag 2014^83^ | 2 | KSA | Egyptian school nurses AE toward sex relationship education | School nurses: KA towards sex education |
| Saleh 2014^140^ | 6 *^b^* | Egypt | Awareness of reproductive health/HIV issues and sources of knowledge | General population: Knowledge towards HIV/AIDS and SRH |
| Othman 2014^141^ | 6 | Iraq | HIV/AIDS knowledge among high school students; association between socio-demographics and HIV/AIDS knowledge | High school students: Knowledge towards HIV/AIDS |
| Akhu-Zaheya 2015^142^ | 8 | Jordan | Sexual concerns/counseling of Muslim cardiac patients; from whom do they wish to receive sexual counselling information; is gender difference a barrier? | Arab-Muslim patients with cardiac problems: attitude towards SRH service. Health care system: availability |
| AlMuzaini 2015^143^ | 6 | Kuwait | HIV/AIDS KA of dental assistants at University Dental Center/MoH hospitals; if intervention needed to provide HIV/AIDS information to them | Dental assistants: KA towards HIV/AIDS |
| Himmich 2015^67^ | 6 | Morocco | HIV testing/counselling community-based intervention for truck drivers | Truck drivers: Knowledge towards HIV/AIDS |
| Aunon 2015^70^ | 6 | Lebanon | factors influencing sexual risk behaviors and HIV testing among MSW | Male Sex Workers: KA towards HIV/AIDS |
| Abdelrahman 2015^55^ | 6 | Egypt | Obstacles health care workers face in providing care for PLWHA | People Living with HIV: attitudes, stigmatization |
| Premadasa 2015^32^ | 6 | Kuwait | Dental students’ knowledge of HIV/AIDS transmission/oral manifestations; attitudes towards obligations to treat patients | Dental students: KA towards HIV/AIDS |
| Memish 2015^46^ | 6 | KSA | HCPs knowledge and stigmatizing attitudes towards HIV/ PLWHA | Physicians: Knowledge and stigmatization towards HIV/AIDS |
| Ghazeeri 2016^73^ | 5 | Lebanon | KAA of fertility preservation among oncologists/ clinical practitioners | Oncologists: KA towards fertility preservation |
| Haroun 2016^144^ | 6 | UAE | HIV/AIDS KA among a wide group of university students | University Students: KA towards HIV/AIDS |
| Hamdan-Mansour 2016^90^ | 3 | Jordan | KAP towards family planning of Jordanian men | Men: KA towards family planning |
| Lohiniva 2016^69^ | 6 | Egypt | HIV stigma-reduction intervention in healthcare setting in Egypt/MENA | HCPs: Stigmatization and attitude towards HIV/AIDS |
| Lohiniva 2016^145^ | 6 | Egypt | Perceived HIV stigma by community members toward PLWH/physicians associated with HIV; stigma reduction guidelines for HIV referral hospitals | People Living with HIV: stigmatization |
| Salameh 2016^84^ | 2 | Lebanon | Attitudes of sexuality/its practice of university students and their correlates | University students: KA towards sexuality |
| Sexty 2016^146^ | 5 | Jordan | Cross-cultural differences in fertility specific quality of life of infertile couples in Germany, Hungary and Jordan who attend fertility centers | General population: attitude towards fertility services |
| Arheiam 2017^47^ | 6 | KSA | Assess intended refusal of recent graduates from 3 Arab dental schools to treat HIV patients; factors associated with intention | Dentists: KA towards HIV/AIDS |
| Ashry 2017^147^ | 6 | Egypt | HRQOL of healthy children/adolescents of HIV-positive/ negative parents | Healthy children and adolescents of HIV-Positive parents: stigmatization |
| Kulane 2017^40^ | 6 | Somalia | Experiences of people with HIV; resilience in access to care/ social support | People Living with HIV: KA towards HIV/AIDS |
| Laraqui 2017^42^ | 6 | Morocco | KAP of seafarers in relation to STI and HIV/AIDS | Seafarers: KA towards STIs and HIV/AIDS |
| Melaibari 2017^78^ | 2 | KSA | KAP of University students toward the national PMS program | University Students: KA towards premarital screening |
| Spindler 2017^148^ | 3 | Jordan | Desk review of USAID programs in Jordan; interviews with reproductive health stakeholders | General population: KA towards family planning |
| Abu Ali 2018^81^ | 8 *^b^* | Jordan | Nurse knowledge about ED in cardiac patients | Nurses: Knowledge and attitudes towards Erectile dysfunction assessment |
| Elgalib 2018^63^ | 6 | Oman | Impact of MDT approach in tertiary HIV clinic; evaluate VL changes of infected patients after introducing the approach | Healthcare system: Program for HIV |
| Alwafi 2018^51^ | 6 | KSA | HIV/AIDS KA among general population | General population: KA towards HIV/AIDS |
| Kabbash 2018^54^ | 6 | Egypt | Stigmatized attitude among health care providers toward PLWH | HCPs: Stigmatization and attitude towards HIV/AIDS |
| Khamis 2018^61^ | 6 | Lebanon | Analysis of activity reports of NAP in Syria to describe HIV by mode of transmission; monitor how war affected HIV surveillance/ VCT | Healthcare system: Program for HIV |
| Elmahy 2018^85^ | 2 *^c^* | Egypt | Sexual/ psychological behavior of gay population | Gays: risk behaviour |
| Alhasawi 2019^24^ | 6 *^b^* | Kuwait | High school students KAA about HIV/AIDS | Senior High School Students: KA towards HIV/AIDS |
| Bashir 2019^57^ | 6 | Sudan | Perspectives of employees/HCP working in HIV prevention in Sudan and Yemen of challenges/facilitating factors faced by HIV prevention programs | Healthcare system: Quality of programs for HIV |
| Al Tall 2020^52^ | 6 | Jordan | HIV patient barriers/supporting factors for adherence; needs for pharmaceutical care services | People Living with HIV: attitude to treatment |
| Bdair 2020^82^ | 8 | KSA | Jordanian nurses’ KAP and beliefs toward sexual health assessment of patients CAD | Nurses: knowledge, attitude and training SRH services |
| Farsi 2020^34^ | 6 | KSA | Knowledge about HPV, vaccination, related oropharyngeal cancer (OPC); vaccine acceptability among dental undergraduates | Dental Students: Knowledge of HPV |
| Bein 2020^60^ | 6 | African countries | Relationship between healthcare spending and health status (mortality rate, maternal death, HIV prevalence, tuberculosis) | Healthcare system: Cost of programs for HIV |
| Elgalib 2020^58^ | 6 | Oman | HIV care cascade in Oman 2015-2018 | Healthcare system: Program for HIV |
| Zaidouni 2020^149^ | 5 | Morocco | Experiences/needs of Moroccan infertile couples in ART | Healthcare system: Availability of ART |
| Shah 2020^150^ | 6 | Oman | HIV-related KAP among healthcare workers | HCPs: KA towards HIV/AIDS |
| Naal 2020^87^ | 2 | Lebanon | HCPs attitudes and behaviors toward LGBT | HCPs: KA towards LGBT |
| Alshehri 2021^39^ | 6 | KSA | KA and awareness of HPV infection/oropharyngeal cancer; vaccination; HPV prevalence; education effect on knowledge/ awareness of HPV infections | General population: KA towards HIV/AIDS |
| Farsi 2021^33^ | 6 | KSA | Knowledge of HPV, vaccine acceptability of male medical students | Male medical students: Knowledge of HPV |
| Gausman 2021^86^ | 2 | Jordan | provider attitudes towards youth-friendly SRH services | HCPs: attitude towards SRH services |
| Sait 2021^151^ | 3 | KSA | KA and perceptions of contraception/ family planning among males | Men: KA towards family planning |
| Karim 2021^152^ | 3 | KSA | Men’s KA and barriers about emergency contraception | General population: Knowledge and attitude towards family planning |
| Maatouk 2021^68^ | 6 | Lebanon | Effectiveness of HIV self-tests (HIVST); discuss how HIVST success impacted via comparison of services pre-COVID-19 and during COVID-19 | Men having Sex with Men: KA towards HIV/AIDS |
| Marih 2021^62^ | 6 | Morocco | Missed opportunities for HIV testing in patients newly diagnosed with HIV | Healthcare system: Quality of programs for HIV |
| Barakat 2022^93^ | 3 | Jordan | Pharmacist KP toward male oral contraceptive pills | Pharmacists: KA towards OCP |
| Barry 2022^59^ | 6 | KSA | Healthcare/ treatment costs of caring for PLWH at tertiary care hospitals | Healthcare system: Program for HIV |
| Kapoor 2022^153^ | 2 *^c^* | Jordan | SRH of unmarried youth encounters and services provided | HCPs: Knowledge and training for SRH assessment |

Arranged in ascending order of year of publication; Due to word limit, only the first author is cited; Some articles addressed an additional secondary domain, namely: *^a^* fertility care, *^b^* comprehensive education & information, *^c^* prevention & control of HIV & other sexually transmissible infections, or *^d^* contraception counselling & provision.

Sexual & reproductive health domains: 1 Antenatal, intrapartum and postnatal care; 2 Comprehensive education and information; 3 Contraception counselling and provision; 4 Gender-based violence prevention, support and care; 5 Fertility care; 6 Prevention and control of HIV and other sexually transmissible infections; 7 Safe abortion care; 8 Sexual function and psychosexual counselling.

AE=attitudes & experiences. ART=Assisted Reproductive Technology. CAD=coronary artery diseases. D=Domain. ED=erectile dysfunction. FGM=female genital mutilation. FSW=female sex workers. HBV=hepatitis B virus. HCPs=health care providers. HCV=hepatitis C virus. HPV=human papilloma virus. HRQOL=health-related quality of life. KA=knowledge & attitudes. KAA=knowledge, awareness & attitude. KAb=knowledge, attitudes & behaviors. KAB=knowledge, attitudes & beliefs. KABP=knowledge, attitudes, beliefs & practices. KAP=Knowledge, attitude & practice. KAS=knowledge, attitude & satisfaction. KP=knowledge & perceptions. KSA=kingdom of Saudi Arabia. LGBT=lesbian, gay, bisexual, and transgender. MENA=Middle East and North Africa. MoH=Ministry of Health. MSM=men who have sex with men. NAP=National AIDS Program. PCC=primary care clinic. PLWH=people living with HIV. PLWHA=people living with HIV and AIDS. PMS=premarital screening. OIDP=oral impacts on daily performances. RHS=reproductive health services. SRH=sexual & reproductive health. STI=sexually transmitted infections. TB=tuberculosis. UAE=United Arab Emirates. VCT=voluntary counselling and testing. VL=Viral load.
